# Distinct characteristics and prognosis of IgA nephropathy patients with nephrotic syndrome: a propensity score-matched cohort study

**DOI:** 10.3389/fmed.2024.1344219

**Published:** 2024-02-19

**Authors:** Yuanyuan Jiang, Pei Chen, Wenjing Zhao, Lijun Liu, Sufang Shi, Jicheng Lv, Hong Zhang

**Affiliations:** ^1^Renal Division, Department of Medicine, Peking University First Hospital, Beijing, China; ^2^Institute of Nephrology, Peking University, Beijing, China; ^3^Department of Nephrology, Beijing Hospital of Traditional Chinese Medicine, Capital Medical University, Beijing, China

**Keywords:** IgA nephropathy, nephrotic syndrome, proteinuria, hypoalbuminemia, complement

## Abstract

**Introduction:**

IgA nephropathy (IgAN) is the most prevalent primary glomerulonephritis globally. While nephrotic syndrome (NS) is uncommon in IgAN, its significance remains unclear.

**Methods:**

We conducted a retrospective analysis of 170 IgAN patients, classifying them into NS (*n* = 85) and non-NS (*n* = 85) groups. Our study aims to compare their clinical characteristics, treatment responses, and prognoses. Patients were selected based on renal biopsy from 2003 to 2020. Propensity score matching ensured comparability. Clinical, pathological, and immunological data were analyzed. Composite endpoints were defined as end-stage kidney disease (ESKD) or a 30% decline in estimated glomerular filtration rate (eGFR).

**Results:**

NS patients showed higher eGFR (74.3 ± 36.8 vs. 61.5 ± 33.6 mL/min.1.73 m^2^, *p* = 0.02), severe hematuria (35.0 (4.7,147.5) vs. 4.0 (1.8,45,0) cells/μl, *p* < 0.001), severe foot process effacement (*p* = 0.01), and lower C3 levels (1.0 ± 0.3 vs. 1.1 ± 0.2 g/L, *p* = 0.03). In contrast, the non-NS group had higher BMI (24.3 ± 4.0 vs. 26.8 ± 3.7 kg/m^2^, *p* < 0.001) and elevated serum uric acid levels (376 (316,417) vs. 400 (362, 501) mmol/L, *p* = 0.001), suggesting metabolic factors might contribute to their condition. Both groups exhibited similar MESTC scores. NS patients had higher complete remission rates (26.2% vs. 14.1%, *p* = 0.04). Cox regression revealed NS independently associated with a higher risk of composite endpoints (HR = 1.97, 95% CI 1.05–3.72, *p =* 0.04). Linear mixed models did not show significant eGFR trajectory differences.

**Discussion:**

This study has established that IgAN patients with NS exhibit distinct characteristics, including active disease and increased complement activation. NS is independently associated with a poorer prognosis, emphasizing the need for targeted interventions in this subgroup.

## 1 Introduction

IgA nephropathy (IgAN) is the most common primary glomerulonephritis worldwide, particularly in Asian countries (1, 2). It is the most common cause of end-stage kidney disease (ESKD) in adolescents (3). About 30–40% of the patients would develop ESKD within 20–30 years after the diagnosis of IgAN (1). The clinical presentation of IgAN is highly individualized, and nephrotic syndrome (NS) is relatively uncommon in IgAN, except in cases where patients exhibit the pathological features of minimal-change disease (MCD) and IgA deposition (4). When compared to other pathological types of glomerulopathy that present with NS, such as membranous nephropathy and MCD, IgAN patients rarely develop hypoalbuminemia, even when they exhibit nephrotic-range proteinuria (5). The incidence of IgAN complicated by NS is reported to occur in only 5–14.7% of cases (6–9).

The significance of proteinuria levels in guiding therapeutic decisions for IgAN cannot be overstated (10). In accordance with KDIGO guidelines, the treatment approach for IgAN patients with NS is contingent upon pathological findings. Specifically, kidney biopsies demonstrating mesangial IgA deposition along with light and electron microscopy features consistent with MCD should be managed similarly to MCD. Patients whose kidney biopsies reveal coexistent features of mesangioproliferative glomerulonephritis (MPGN) should receive treatment akin to individuals at high risk of progressive chronic kidney disease (CKD), even with maximal supportive care (11). However, it’s noteworthy that the KDIGO guidelines do not provide specific recommendations for managing IgAN patients with nephrotic syndrome who exhibit pathology other than MCD or MPGN. Additionally, it’s worth noting that IgAN patients with NS have typically been excluded from randomized control trials aimed at exploring effective strategies to manage IgAN. This exclusion has left a significant gap in our understanding and treatment options for IgAN patients with NS. Moreover, the pathogenesis of IgAN with NS remains unclear, limiting targeted therapy for these patients.

Consequently, there is a pressing need to conduct a comparative analysis of the clinical manifestations, biomarker levels, treatment responses, and prognoses of IgAN patients with NS and those with nephrotic-range proteinuria but no hypoalbuminemia. Such a comparison is essential for tailoring individualized therapy strategies for these two distinct patient groups.

## 2 Materials and methods

### 2.1 Study design and patients

We conducted a retrospective analysis of 1995 patients diagnosed with IgAN based on renal biopsy at Peking University First Hospital from 2003 to 2020. Inclusion criteria comprised: Patients with a confirmed diagnosis of IgAN through renal biopsy; Patients exhibiting nephrotic-range proteinuria, defined as 24-h urinary total protein (UTP) ≥ 3.5 g/24 h (*n* = 318). We excluded the following cases: Patients with renal biopsy results suggesting IgAN coexisting with MCD (*n* = 19); Cases with pathological findings indicating mild mesangial hyperplasia with podocytopathy (*n* = 4); Patients presenting secondary causes of mesangial IgA deposits or IgAN, such as those with Henoch–Schönlein purpura, systemic lupus erythematosus, liver cirrhosis, or diabetes mellitus (*n* = 17); Cases with fewer than eight glomeruli available for evaluation (*n* = 1); Individuals with IgAN combined with other kidney diseases, such as diabetic nephropathy (*n* = 8); Patients with a baseline estimated glomerular filtration rate (eGFR) ≤ 15 mL/min/1.73 m^2^ (*n* = 6). Ultimately, 263 patients were enrolled. In our study, we classified the patients into two distinct groups based on their serum albumin levels: the NS group, consisting of individuals with serum albumin levels below 30 g/L, and the non-NS group, comprising those with serum albumin levels of 30 g/L or higher. To ensure comparability between these two groups, we performed propensity score matching with a 1:1 ratio, focusing on matching their UTP levels. This matching process resulted in a total of 85 patients in each group. Subsequently, we conducted a comprehensive retrospective analysis of the clinical, pathological, and immunological data of these patients. This study received approval from the Ethics Committee at Peking University First Hospital, and all participating patients provided informed consent.

### 2.2 Data collection

The relevant clinical parameters were retrospectively collected both at the time of biopsy and during the follow-up period. These parameters included age at the time of biopsy, gender, body mass index (BMI), systolic blood pressure, diastolic blood pressure, mean arterial pressure (MAP), serum creatinine, eGFR, serum uric acid levels, UTP, albumin-to-creatinine ratio (ACR) microscopic hematuria, serum IgA and C3, low-density lipoprotein (LDL), triglyceride (TG), and total cholesterol (TCHO) levels. Blood pressure values were consistently expressed in millimeters of mercury (mmHg). The eGFR was computed using the Chronic Kidney Disease Epidemiology Collaboration (CKD-EPI) formula (12). Additionally, we retrospectively gathered the Oxford Pathology Score at the time of renal biopsy (13). This scoring was conducted by two pathologists, namely WSX and SSF. Mesangial C3 deposits were classified into five groups: 0, 1+, 2+, 3+, and 4+. We categorized the extent of foot process effacement in podocytes into three grade groups, which were defined as follows: (1) mild, (2) moderate, and (3) diffuse.

Furthermore, we conducted a retrospective data collection regarding patients’ treatment, which included the use of corticosteroids, other immunosuppressive agents (such as cyclophosphamide, cyclosporin, tacrolimus, mycophenolate mofetil, and leflunomide), as well as Renin-angiotensin-aldosterone system inhibitors (ACEI/ARB). We also documented the responsiveness of patients to the prescribed treatment. Furthermore, data on the follow-up duration and whether patients reached the defined endpoint were also included in our analysis.

### 2.3 Definitions

Hypoalbuminemia was defined as serum albumin <30 g/L. Nephrotic-range proteinuria was defined as proteinuria exceeding 3.5 g/d. NS was characterized by nephrotic-range proteinuria (>3.5 g/d), hypoalbuminemia (<30 g/L), hypercholesterolemia, and edema. Complete remission (CR) was defined as the absence of proteinuria (UTP < 0.3 g/d), accompanied by the disappearance of edema, normalization of all biochemical parameters, and no worsening of renal function. Partial remission (PR) was defined as a reduction of more than 50% in UTP from baseline to less than 3.5 g/d. Spontaneous Remission (SR) denoted complete remission of proteinuria without the use of corticosteroids or other immunosuppressive agents. No response (NR) was defined as a reduction of less than 50% in proteinuria or an increase in proteinuria, with or without renal deterioration. The composite endpoint was defined as either the development of ESKD or a 30% decline in eGFR throughout the follow-up period. ESKD was characterized by either the requirement for dialysis or an eGFR of ≤15 mL/min/1.73m^2^.

### 2.4 Detection of immunological data

Plasma samples were collected from the patients before renal biopsy and were subsequently stored at −80°C until further analysis. The circulating galactose-deficient IgA1 (Gd-IgA1) was quantified using an enzyme-linked immunosorbent assay (ELISA) based on a previously established protocol (14). C3 was measured by rate turbidimetry and rate nephelometry in Beckman Image 800 nephelometer (Beckman Coulter Inc., CA).

### 2.5 Statistical analysis

Measurement data that followed a normal distribution were presented as mean ± standard deviation (SD). Differences between the two groups were assessed using Student’s *t*-test. For measurement data that did not follow a normal distribution, the data were expressed as median (interquartile range), and the comparison between groups was conducted using the Mann–Whitney U test. Dichotomous data were presented as frequency (constituent ratio), and group comparisons were performed using the Pearson chi-squared test. We examined the odds ratio (OR) and the corresponding 95% confidence intervals (CI) in logistic regression models to assess the relationship between baseline characteristics and proteinuria remission. The probability of renal survival was evaluated using Kaplan–Meier survival curves, and group comparisons were performed with the log-rank test. To identify independent factors associated with the development of endpoints, we utilized a Cox proportional hazards model. This model was adjusted for demographic characteristics, including age and sex, prognostic factors of IgAN such as eGFR, proteinuria, MAP, Oxford classification scores, and the use of immunosuppressive therapies. The Schoenfeld residuals test was employed to assess the proportional hazards assumption. The eGFR slope during follow-up was calculated using a mixed model with repeated measures. This model included group, time, and the interaction of group and time as fixed factors. Additionally, baseline eGFR, UTP, MAP, age, sex, and Oxford MESTC classification were included as fixed factors. Participant and intercept were considered as random factors in the analysis. UTP was normalized using Z-scores. A significance level of *p* < 0.05 was considered statistically significant. The statistical analysis was conducted using SPSS version 22.0 software and Stata version 16.

## 3 Results

### 3.1 Comparison of clinical and histopathological characteristics

**Figure 1:**
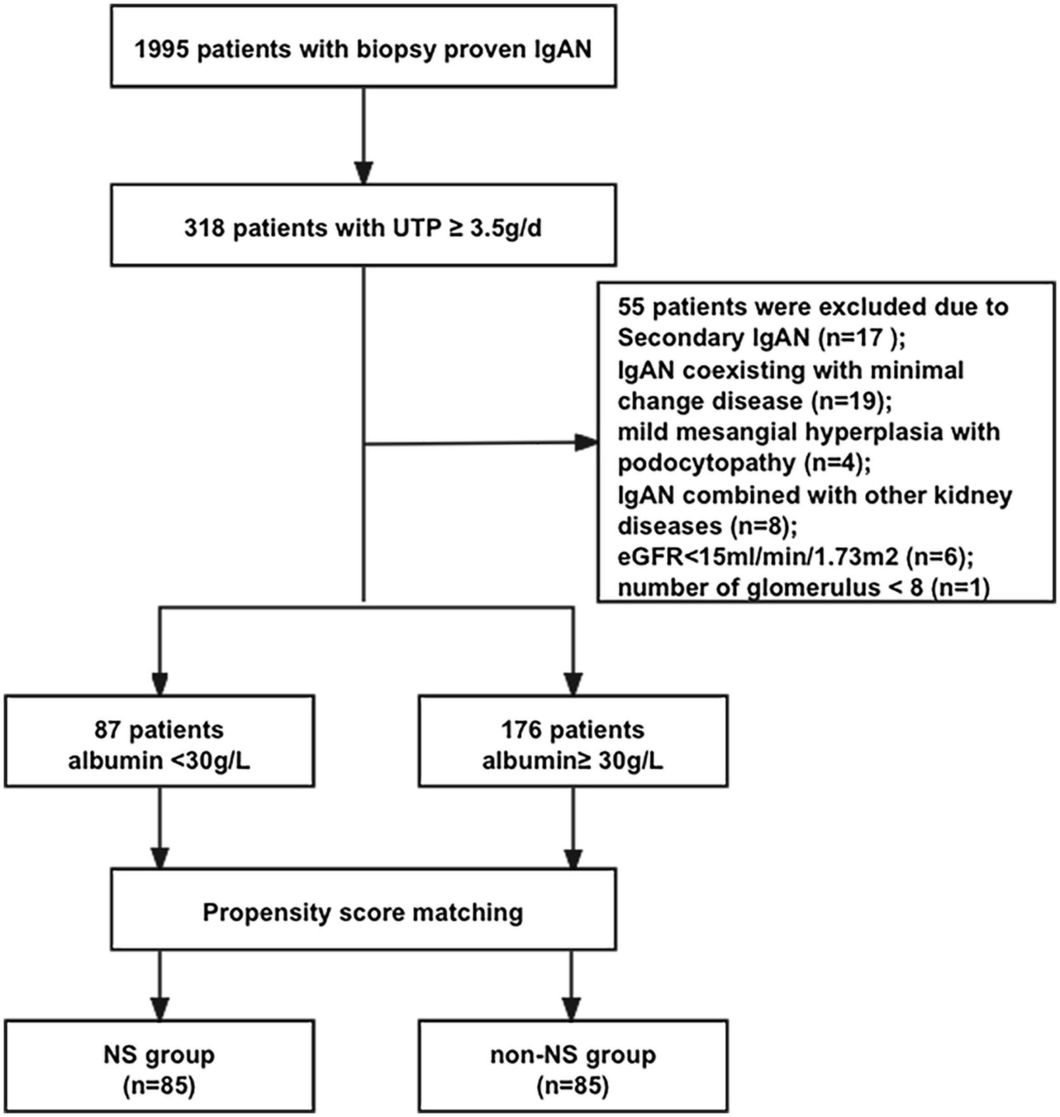
The flow chart for patients’ selection.

A flow diagram illustrating the patient sample and exclusions is presented in Figure 1. Out of a total of 263 patients (13.2%) with proteinuria exceeding 3.5 g/d, 87 patients (4.4%) had NS, while 176 patients (8.8%) displayed nephrotic-range proteinuria with normal serum albumin levels. Among patients with proteinuria exceeding 3.5 g/d, 33.1% had NS. Patients with NS exhibited significantly higher levels of UTP (5.88 (4.26, 8.08) vs. 4.62 (3.97, 6.08) g/d, *p* < 0.001) and ACR (3.45 (2.40, 4.14) vs. 1.96 (1.48, 3.12) mg/g, *p* < 0.001) compared to patients with nephrotic-range proteinuria and normal serum albumin. We performed propensity score matching (1:1) based on UTP levels, resulting in the establishment of 85 patients with nephrotic-range proteinuria and normal serum albumin (non-NS group) who had comparable UTP levels to the 85 patients with NS (NS group). Subsequently, a total of 170 IgAN patients (85 patients in the NS group and 85 patients in the non-NS group) were included in the analysis.

The baseline characteristics of all patients from both groups are presented in Table 1. In terms of renal function, the NS group had higher eGFR levels (74.3 ± 36.8 vs. 61.5 ± 33.6 mL/min.1.73 m^2^, *p =* 0.02) compared to the non-NS group, indicating better renal function. Microscopic hematuria was more pronounced in the NS group (35.0 (4.7, 147.5) vs. 4.0 (1.8, 45,0) cells/μl, *p <* 0.001) compared to the non-NS group. The NS group also exhibited higher levels of LDL and TCHO (4.0 (3.1, 5.1) vs. 3.2 (2.3, 4.1) mmol/L, *p <* 0.001; 6.8 (5.6, 8.7) vs. 6.0 (4.9, 7.0) mmol/L, *p* < 0.001), indicative of differences in lipid profiles. On the other hand, the non-NS group had higher BMI (24.3 ± 4.0 vs. 26.8 ± 3.7, *p* < 0.001) and elevated serum uric acid levels (376 (316, 417) vs. 400 (362, 501) mmol/L, *p =* 0.001) compared to the NS group. There were no significant differences between the two groups in terms of age, gender, blood pressure, history of precursor infection, and gross hematuria. Regarding pathological characteristics, both groups showed similar MESTC scores. However, the NS group displayed more diffuse foot process effacement in electron microscopy (*p* = 0.01).

**Table 1:** Comparison of clinical and pathological characteristics.

Characteristics	NS group (*n* = 85)	Non-NS group (*n* = 85)	*p* value
Age (years)	35.3 ± 17.2	35.9 ± 12.9	0.80
Male (%)	42 (49.4%)	52 (61.2%)	0.12
History of precursor infection (%)	40 (56.3%)	39 (58.2%)	0.82
History of gross hematuria (%)	19 (26.8%)	16 (23.9%)	0.70
MAP (mmHg)	95 (90, 103)	97 (93, 107)	0.15
BMI (kg/m^2^)	24.3 ± 4.0	26.8 ± 3.7	<0.001
eGFR (ml/min/1.73m^2^)	74.3 ± 36.8	61.5 ± 33.6	0.02
CKD stage (%)			
Stage 1	26 (30.6%)	21 (24.7%)	0.39
Stage 2	23 (27.1%)	18 (21.2%)	0.37
Stage 3	29 (34.1%)	30 (35.3%)	0.87
Stage 4	7 (8.2%)	16 (18.8%)	0.04
UTP (g/d)	5.8 (4.3, 7.7)	5.7 (4.3, 7.1)	0.66
Serum albumin (g/L)	24.3 ± 4.0	35 ± 5.2	<0.001
UA (mmol/L)	376 (316, 417)	400 (362, 501)	0.001
Serum IgA (g/L)	2.9 ± 1.0	2.9 ± 1.1	0.62
Serum C3 (g/L)	1.0 ± 0.3	1.1 ± 0.2	0.03
Gd-IgA1(IU/ml)	79.2 ± 9.2	79.6 ± 11.3	0.81
Gd-IgA1/C3	82.9 ± 26.7	75.7 ± 21.1	0.05
Microscopic Hematuria (/ul)	35.0 (4.7, 147.5)	4.0 (1.8, 45,0)	<0.001
LDL (mmol/L)	4.0 (3.1, 5.1)	3.2 (2.3,4.1)	<0.001
TG (mmol/L)	2.1 (1.3, 3.2)	2.5 (1.8, 3.2)	0.15
TCHO (mmol/L)	6.8 (5.6, 8.7)	6.0 (4.9, 7.0)	<0.001
Oxford classification			
M 0/1	31/54	33/52	0.75
E0/1	32/53	40/45	0.21
S0/1	35/50	33/52	0.75
T0/1/2	41/28/16	36/34/15	0.63
C0/1/2	21/35/29	26/40/19	0.23
Mesangial C3 deposits (0 to 2+/3+ to 4+)	53/32	52/33	0.88
Severity of foot process effacement (mild/moderate/diffuse)^†^	0/22/57	1/34/34	0.01

NS, nephrotic syndrome; MAP, mean arterial pressure; BMI, body mass index; Gd-IgA1, galactose-deficient IgA1; UA, uric acid; LDL, low-density lipoprotein; TG, triglyceride; TCHO, Total Cholesterol. ^†^Six patients in the NS group and sixteen patients in the non-NS group either had no glomeruli found in electron microscopy or only had sclerotic glomeruli identified in electron microscopy.

### 3.2 Immunological data

**Figure 2:**
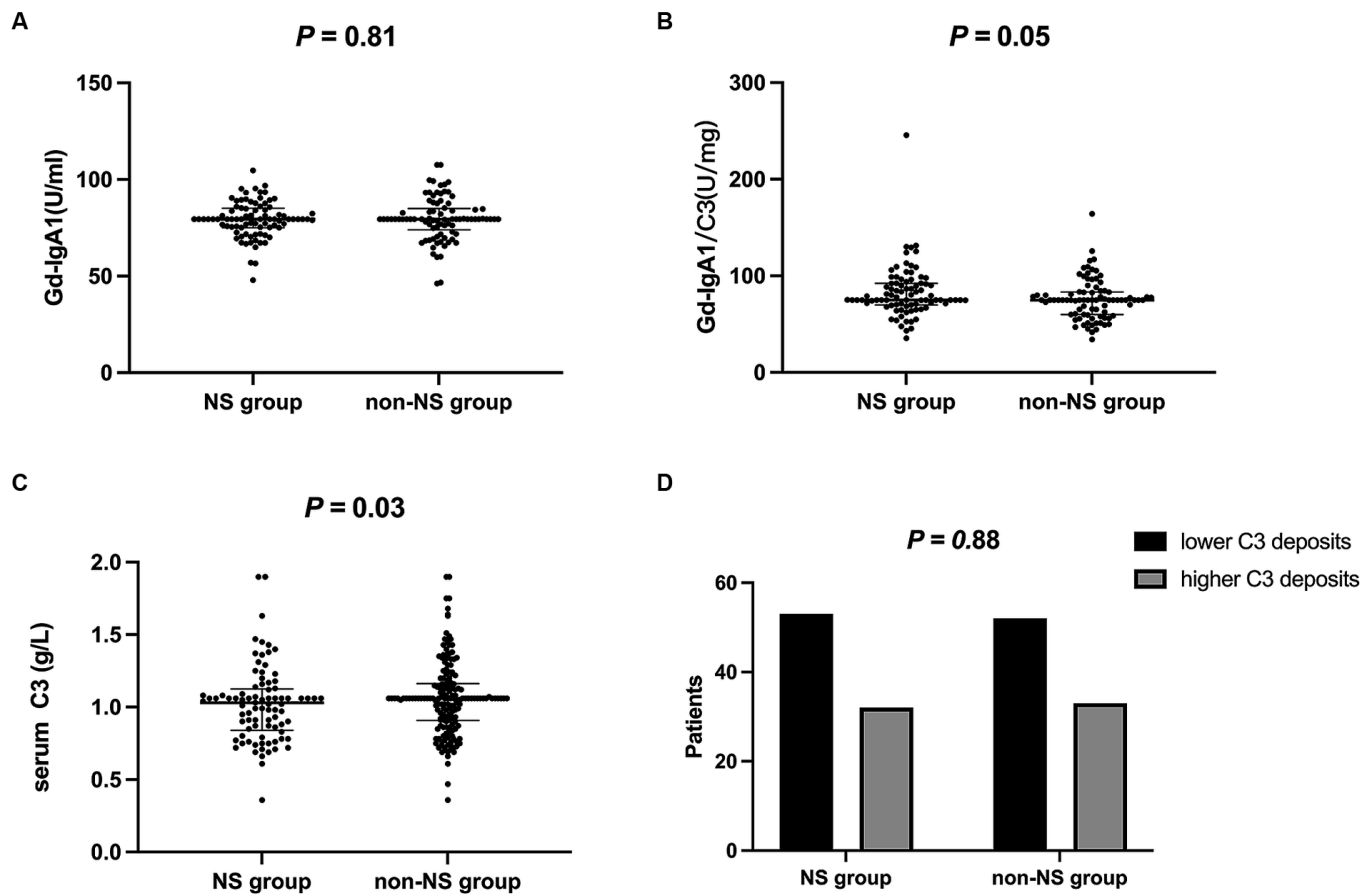
Immunological data of two groups. **(A)** Gd-IgA1 levels were comparable between the groups (*P* = 0.81); **(B)** Gd-IgA1/C3 ratio showed no significant difference between the two groups (*P* = 0.05); **(C)** The NS group showed lower C3 levels (*P* = 0.03); **(D)** C3 deposits were comparable between the groups (*P* = 0.88). lower C3 deposits, 0 to 2+; higher C3 deposits, 3+ to 4+. Gd-IgA1, galactose-deficient IgA1; NS, nephrotic syndrome.

The NS group showed lower C3 levels (1.0 ± 0.3 vs. 1.1 ± 0.2 g/L, *p* = 0.03). However, no significant differences were observed between the groups in terms of C3 deposits in renal histology under immuno-fluorescence when comparing lower-grade (0 to 2+) and higher-grade (3+ to 4+) C3 deposits (*p* = 0.88). Gd-IgA1 levels were comparable between the groups (79.2 ± 9.2 vs. 79.6 ± 11.3 U/mL, *p* = 0.81). Similarly, the Gd-IgA1/C3 ratio showed no significant difference between the two groups (82.9 ± 26.7 vs. 75.7 ± 21.1, *p* = 0.05) (Figure 2).

### 3.3 Treatment and treatment responsiveness

The treatment and treatment responsiveness are detailed in Table 2. In terms of treatment approaches, the non-NS group had a higher percentage of patients solely using RAASi (5.9% vs. 17.6%, *p* = 0.02). Patients with NS showed a higher proportion of corticosteroid use (92.9% vs. 76.5%, *p* = 0.003). A subset of patients in both groups received a combination of corticosteroids and immunosuppressive agents, and these groups exhibited comparable percentages (71.8% vs. 57.6%, *p* = 0.05). Among patients who received immunosuppressive agents, cyclophosphamide was the most commonly employed, followed by mycophenolate mofetil.

**Table 2:** Comparison of treatment and responsiveness in two groups.

Characteristics	NS group (*n* = 85)	Non-NS group (*n* = 85)	*p* value
**Treatment**			
RAASi only (%)	5 (5.9%)	15 (17.6%)	0.02
Corticosteroid (%)	79 (92.9%)	65 (76.5%)	0.003
Corticosteroid combined with Immunosuppressive agents (%)	61 (71.8%)	49 (57.6%)	0.05
Cyclophosphamide	33 (38.8%)	26 (30.6%)	0.59
Mycophenolate mofetil	17 (20%)	11 (12.9%)	0.21
Ciclosporin	16 (18.8%)	8 (9.4%)	0.08
Tacrolimus	3 (3.5%)	5 (5.9%)	0.47
Leflunomide	11 (12.9%)	13 (15.3%)	0.66
Tripterygium wilfordii	11 (12.9%)	15 (17.6%)	0.39
**Proteinuria remission**			
CR/PR/NR	24/38/23	12/53/20	0.04
CR + PR/NR	62/23	65/20	0.72

CR, Complete remission; PR, Partial remission; NR, no remission or disease progression; RAASi, Renin—angiotensin—aldosterone system inhibitors.

After a median follow-up period of 41 months (ranging from 12 months to 192 months), the NS group showed a higher proportion of CR in proteinuria (26.2% vs. 14.1%, *p* = 0.04). Specifically, 2 patients in the NS group and 4 patients in the non-NS group experienced SR. However, when considering both CR and PR together, the proteinuria remission rates between the two groups were comparable (72.9% vs. 76.5%, *p* = 0.72). We also calculated the time-average proteinuria during follow-up, and the results showed no significant difference between the two groups (1.44 (0.75, 3.40) vs. 1.76 (1.23, 2.38) g/d, *p* = 0.67). To investigate the factors associated with CR in NS patients, a logistic regression analysis was conducted (Table 3). The results indicated that several variables were linked to a reduced likelihood of CR in a model that adjusted for age, gender, baseline GFR, and MAP. These variables included M lesion (odds ratio (OR) 0.08, 95% confidence interval (CI) 0.01–0.57, *p* = 0.01), E lesion (OR 0.16, 95% CI 0.03–0.95, *p* = 0.04), and C2 score (OR 0.02, 95% CI 0.01–0.60, *p* = 0.02).

**Table 3:** Univariate and multivariate logistic regression analysis of factors influencing complete remission in IgA nephropathy with nephrotic syndrome.

Characteristics	Univariate			Multivariate		
	OR	95% CI	*p*	OR	95% CI	*p*
Age (per year)	0.93	0.83 to 1.05	0.30	1.02	0.94 to 1.09	0.69
Gender (male)	1.96	0.74 to 5.17	0.17	12.17	1.49 to 99.04	0.02
eGFR (ml/min/1.73m^2^)	1.02	1.01 to 1.04	0.003	1.03	0.99 to 1.07	0.10
MAP (mmHg)	0.97	0.93 to 1.01	0.20	1.02	0.94 to 1.10	0.66
Oxford classification						
M1	0.36	0.14 to 0.96	0.04	0.08	0.01 to 0.57	0.01
E1	0.12	0.18 to 1.22	0.46	0.18	0.02 to 1.56	0.12
S1	0.18	0.06 to 0.50	0.001	0.16	0.03 to 0.95	0.04
T1	0.29	0.09 to 0.92	0.04	0.50	0.05 to 5.27	0.56
T2	0.09	0.01 to 0.71	0.02	0.13	0.003 to 5.45	0.28
C1	0.32	0.10 to 1 0.03	0.06	1.16	0.20 to 6.71	0.87
C2	0.07	0.01 to 0.35	0.002	0.02	0.01 to 0.60	0.02
Corticosteroid or immunosuppressive agents	0.57	0.09 to 3.64	0.55	0.06	0.01 to 4.10	0.19

MAP, mean arterial pressure; eGFR, estimated glomerular filtration rate; CI, confidence interval; OR, odds ratio.

### 3.4 Prognosis analysis

**Figure 3:**
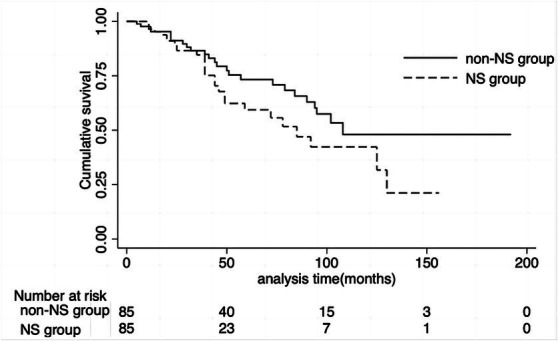
Cumulative renal survival rate of NS group and non-NS group. The cumulative survival rate until the composite endpoint was similar in both groups (log-rank test: *P* = 0.12). NS, nephrotic syndrome.

**Figure 4:**
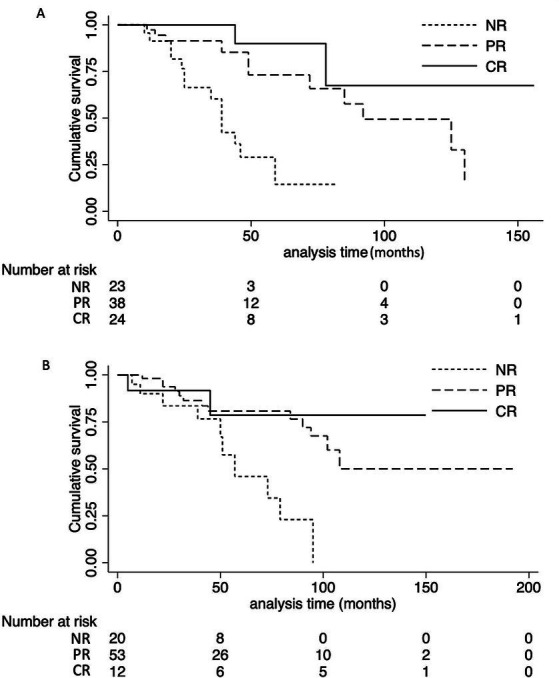
Effect of clinical response on the cumulative renal survival rate of NS group and non-NS group. **(A)** Patients in the NS group attaining CR or PR had a favorable outcome compared with patients with NR (log-rank test: *P* = 0.02). **(B)** Patients in the non-NS group attaining CR or PR had a favorable outcome compared with patients with NR (log-rank test: *P* < 0.001). NS, nephrotic syndrome. NR, not remission; PR, partial remission; CR, complete remission.

Twenty-seven patients (31.8%) in the NS group reached the composite endpoint, while 25 patients (29.5%) in the non-NS group did so. Specifically, fifteen patients (17.6%) in the NS group reached ESKD, while six patients (7.6%) in the non-NS group experienced ESKD. The cumulative survival rate until the composite endpoint was similar in both groups (log-rank test: *p* = 0.12) (Figure 3). As indicated in the Kaplan–Meier survival curves, renal survival differed based on proteinuria remission in both groups (NS group, log-rank test: *p* = 0.02; non-NS group, log-rank test: *p* < 0.001) (Figure 4). We employed the Cox proportional hazards model to evaluate the relationship between the presence of NS and the composite endpoint. In unadjusted analysis, lower baseline eGFR and higher T score were significantly associated with a higher risk of progression (Table 4). After adjusting for traditional risk factors, including age, gender, baseline eGFR, baseline UTP, baseline MAP, METSC scores, and immunosuppressive therapy, the NS group was independently associated with an increased risk of the composite endpoint, with a hazard ratio of 1.97 (95% CI, 1.05 to 3.72; *p* = 0.04). The analysis of the eGFR trajectory over time using a linear mixed-effects model did not reveal a significant difference between the two groups. The difference in the eGFR trajectory over time between the NS group and the non-NS group was −1.28 mL/min/1.73 m^2^ per year (95% CI, −0.67 to 3.32 mL/min/1.73 m^2^ per year, *p* = 0.20).

**Table 4:** Cox proportional hazard model for the primary endpoint in IgA nephropathy patients with nephrotic syndrome.

	Unadjusted		Model 1^a^		Model 2^b^		Model 3^c^	
	HR (95% CI)	*p*	HR (95% CI)	*p*	HR (95% CI)	*P*	HR (95% CI)	*p*
NS or not	1.53 (0.88–2.66)	0.13	1.53 (0.88–2.65)	0.13	2.54 (1.40–4.63)	0.002	1.97 (1.05–3.72)	0.04
Gender (male)	0.84 (0.48–1.47)	0.53	0.82 (0.47–1.45)	0.50	0.67 (0.37–1.22)	0.19	0.62 (0.33–1.17)	0.14
Age (per year)	1.00 (0.98–1.01)	0.63	1.00 (0.98–1.01)	0.66	0.96 (0.94–0.98)	0.001	0.98 (0.95–1.01)	0.12
eGFR (ml/min/1.73m^2^)	0.98 (0.97–0.99)	0.001			0.97 (0.96–0.98)	<0.001	0.98 (0.97–1.00)	0.04
Proteinuria (g/d)	0.93 (0.83–1.04)	0.20			0.90 (0.81–1.01)	0.07	0.91 (0.79–1.04)	0.17
MAP (mmHg)	1.01 (0.99–1.03)	0.45			1.01 (0.98–1.03)	0.60	1.01 (0.99–1.04)	0.42
Oxford classification								
M1	1.27 (0.72–2.24)	0.40					0.63 (0.31–1.29)	0.21
E1	0.98 (0.57–1.69)	0.94					1.07 (0.55–2.08)	0.84
S1	1.96 (1.05–3.68)	0.04					1.26 (0.59–2.66)	0.55
T1	2.86 (1.40–5.84)	<0.001					2.05 (0.87–4.87)	0.10
T2	7.39 (3.50–15.61)	<0.001					5.22 (1.90–14.31)	<0.001
C1	2.59 (1.18–5.71)	0.02					2.14 (0.91–5.02)	0.08
C2	3.03 (1.29–7.09)	0.01					1.95 (0.73–5.20)	0.18
Corticosteroid or Immunosuppressive agents	0.84 (0.48–1.47)	0.53					1.68 (0.48–5.83)	0.42

CI, confidence interval; MAP, mean arterial pressure; eGFR, estimated glomerular filtration rate.

a Model 1 was adjusted for sex and age.

b Model 2 was adjusted for covariates in model 1 plus eGFR, proteinuria, and mean arterial blood pressure.

c Model 3 was adjusted for covariates in model 2 plus Oxford M (mesangial hypercellularity), E (the presence of endocapillary proliferation), S (segmental glomerulosclerosis/adhesion), T (severity of tubular atrophy/interstitial fibrosis), and C (presence of crescent) scores and steroids or other immunosuppressive agents (yes or no). Composite end point was defined as a 30% decline in eGFR or ESKD.

## 4 Discussion

Very few studies have specifically addressed IgAN patients with NS (5–9, 15, 16). It remains uncertain whether IgAN patients with NS exhibit distinct pathogenesis and require different management strategies. To address this knowledge gap, we conducted a propensity score-matched cohort study to delineate the characteristics of IgAN patients with NS. Our study revealed that approximately 4.4% of IgAN patients in our cohort presented with NS. In comparison to IgAN patients with similar levels of proteinuria, those with NS exhibited higher eGFR levels, a greater prevalence of hematuria, and reduced serum C3 levels. When it comes to treatment responsiveness, IgAN patients with NS showed a higher rate of CR in terms of proteinuria. It was observed that patients with higher M, E, and C scores were less likely to achieve complete remission. Additionally, NS was identified as an independent risk factor associated with an increased risk of the composite endpoint in Cox regression analysis.

The prevalence of IgAN with NS in our study was notably lower than what previous research has reported, where the prevalence ranged from 5.0 to 14.7% (6–8). This difference in prevalence can be attributed, in part, to our deliberate exclusion of special forms of IgAN, such as cases where IgAN coexisted with MCD, cases involving mild mesangial hyperplasia with podocytopathy, and IgAN combined with other kidney diseases. In terms of clinical manifestation, our study unveiled those individuals with IgAN and NS exhibited heightened hematuria and lower C3 levels. These outcomes suggest a more pronounced disease activity and elevated complement activation in this subset of patients. Pathologically, those with IgAN and NS demonstrated a more severe foot process effacement, aligning with previous research, thereby signifying podocyte injury in these cases (17, 18). Interestingly, despite the more severe pathological findings, the NS group presented with a superior baseline renal function. This paradox could be attributed to the acute and early nature of IgAN in this group, in contrast to the prolonged and chronic features observed in the non-NS group. Conversely, in the non-NS group, there was a notable presence of higher BMI and elevated serum uric acid levels. This observation implies that metabolic factors, particularly obesity, might play a significant role in the development of nephrotic-range proteinuria in these patients. This aligns with KDIGO guidelines, which recognize that patients presenting with nephrotic-range proteinuria while maintaining normal serum albumin levels often indicate the presence of coexistent secondary focal segmental glomerulosclerosis (FSGS). This association is frequently observed in individuals with conditions like obesity or uncontrolled hypertension, or the context of extensive glomerulosclerosis and tubulointerstitial fibrosis (11). Unfortunately, none of these specific descriptions of secondary FSGS were available in the pathological reports of these patients, thereby limiting the supportive evidence for the hypothesis of pathogenesis in the non-NS group in pathology.

Furthermore, we conducted measurements of Gd-IgA1 and the Gd-IgA1/C3 ratio in this study. These parameters have been previously reported as associated with the pathogenesis and prognosis of IgAN patients (14, 19). Notably, the Gd-IgA1/C3 ratio displayed a higher trend in the NS group, although it did not reach statistical significance (*p* = 0.05). This lack of significance may be attributed, in part, to the sample size, which was not sufficiently large to establish a definitive relationship. The clinical manifestations and biomarker profiles suggested that immune factors may underlie IgAN patients with NS, while metabolic factors could be more influential in the other group of patients.

In our study, the proportion of proteinuria remission differed between IgAN patients with NS and the non-NS group, partly due to a higher CR proportion in the NS group (26.2% vs. 14.1%). In a study by JK Kim et al., CR was reported to occur in as many as 48% of patients, with SR reaching 24% (8). Their treatment responsiveness was notably better than what we observed. This difference could be attributed to their inclusion of IgAN patients with MCD and their restriction to patients with eGFR over 30 mL/min/1.73m^2^. Although the NS group received more positive treatment, as evidenced by a higher proportion undergoing corticosteroid therapy (*p* = 0.003) and a trend towards corticosteroid combined with Immunosuppressive agents (*p* = 0.05), the correlation with kidney progression events persisted. This suggests a poorer prognosis in patients with NS, emphasizing the importance of targeted interventions in this subgroup. Furthermore, the Cox model did not identify active immunosuppressive therapy as an independent protective factor for improved renal prognosis. This result could be attributed to our approach of categorizing patients into whether they used corticosteroid or immunosuppressive agents, rather than stratifying them based on specific treatment combinations, such as corticosteroid alone, corticosteroid combined with immunosuppressive agents, and immunosuppressive agents alone, due to limitations in sample size. Given the lower serum C3 levels observed in IgAN patients with NS, it would be intriguing to explore the potential effectiveness of complement system inhibitors in these individuals, such as humanized monoclonal antibodies against C5, C5a receptor blockers, and peptide inhibitors of C3 (20–22) Conversely, the non-NS group exhibited higher BMI levels. For these patients, it may be worth considering the use of sodium-glucose cotransporter 2 inhibitors (SGLT2i) in their management. SGLT2 inhibitors have been reported to reduce proteinuria and improve prognosis in IgAN (23, 24). Additionally, they have the added benefit of promoting weight loss, making them a potentially valuable option for this group of patients.

The study’s analysis revealed a notable finding related to the poor prognosis of IgAN patients with NS. This finding was primarily derived from Cox regression analysis. However, it’s worth noting that it wasn’t confirmed by linear mixed models. One possible explanation for this lack of confirmation could be the presence of numerous covariates considered in the analysis, potentially exceeding what the sample size could adequately account for. The complexity of the data and the number of variables involved may have limited the linear mixed models’ ability to detect significant differences in this aspect of the study. In order to validate the results of the Cox regression analysis, further research with a larger sample size may be imperative to comprehensively explore kidney outcomes in patients with IgA nephropathy and NS. Previous research that compared IgAN patients with NS to those with typical IgAN concluded that the prognosis for NS in IgAN was unfavorable unless partial remission PR or CR of proteinuria was achieved (8, 15, 16, 25). It’s worth noting that there was a substantial difference in UTP levels between the two groups in those studies. To ensure that UTP levels were comparable, we used propensity score matching, but despite this adjustment, we still found a higher risk of a composite renal outcome in IgAN patients with NS. This emphasizes the challenging nature of NS in IgAN.

Our study has several limitations that should be acknowledged. Firstly, while our sample size was relatively large compared to previous studies, it may still not be sufficient, especially considering the numerous covariates included in the multivariate models. Secondly, when analyzing the therapeutic approaches, we did not evaluate the impact of hydroxychloroquine and SGLT2 inhibitors, both of which have become increasingly relevant in the management of IgAN. Thirdly, we observed lower serum C3 levels in the NS group, indicative of complement activation, but we did not measure serum complement activation fragments such as C3a, C3c, and C4d to confirm our hypothesis and determine the specific complement activation pathway.

In conclusion, our study revealed distinct characteristics in IgAN patients with NS. These patients exhibited higher eGFR levels, heavier hematuria, and reduced serum C3 levels, suggesting more active disease and increased complement activation. Conversely, IgAN patients with nephrotic-range proteinuria but without hypoalbuminemia had higher BMI and elevated serum uric acid levels, indicating potential metabolic factors at play. Importantly, IgAN patients with NS had a worse prognosis compared to those with comparable proteinuria, highlighting the necessity for specialized and targeted interventions in this subgroup of patients.

## Data availability statement

The raw data supporting the conclusions of this article will be made available by the authors, without undue reservation.

## Ethics statement

The studies involving humans were approved by Ethics Committee of Peking University First Hospital. The studies were conducted in accordance with the local legislation and institutional requirements. The participants provided their written informed consent to participate in this study.

## Author contributions

YJ: Investigation, Writing – original draft. PC: Formal analysis, Writing – review & editing. WZ: Validation, Writing – original draft. LL: Data curation, Writing – review & editing. SS: Data curation, Writing – review & editing. JL: Conceptualization, Writing – review & editing. HZ: Conceptualization, Writing – review & editing.
