# Corrigendum: Distinct characteristics and prognosis of IgA nephropathy patients with nephrotic syndrome: a propensity score-matched cohort study

**DOI:** 10.3389/fmed.2024.1400735

**Published:** 2024-04-17

**Authors:** Yuanyuan Jiang, Pei Chen, Wenjing Zhao, Lijun Liu, Sufang Shi, Jicheng Lv, Hong Zhang

**Affiliations:** ^1^Renal Division, Department of Medicine, Peking University First Hospital, Beijing, China; ^2^Institute of Nephrology, Peking University, Beijing, China; ^3^Department of Nephrology, Beijing Hospital of Traditional Chinese Medicine, Capital Medical University, Beijing, China

**Keywords:** IgA nephropathy, nephrotic syndrome, proteinuria, hypoalbuminemia, complement

In the published article, there was an error in the **Abstract** section.

This sentence previously stated:

“We conducted a retrospective analysis of 170 IgAN patients, classifying them into NS (n = 85) and non-NS (n = 5) groups.”

The corrected sentence appears below:

“We conducted a retrospective analysis of 170 IgAN patients, classifying them into NS (*n* = 85) and non-NS (*n* = 85) groups.”

The authors apologize for this error and state that this does not change the scientific conclusions of the article in any way. The original article has been updated.

